# A systematic review of lopinavir therapy for SARS coronavirus and MERS coronavirus—A possible reference for coronavirus disease‐19 treatment option

**DOI:** 10.1002/jmv.25729

**Published:** 2020-03-12

**Authors:** Tian‐Tian Yao, Jian‐Dan Qian, Wen‐Yan Zhu, Yan Wang, Gui‐Qiang Wang

**Affiliations:** ^1^ Department of Infectious Diseases and the Center for Liver Diseases Peking University First Hospital Beijing China; ^2^ The Collaborative Innovation Center for Diagnosis and Treatment of Infectious Diseases, Zhejiang University Hangzhou Zhejiang China; ^3^ Peking University International Hospital Beijing China

**Keywords:** coronavirus, COVID‐19, lopinavir, MERS, SARS

## Abstract

In the past few decades, coronaviruses have risen as a global threat to public health. Currently, the outbreak of coronavirus disease‐19 (COVID‐19) from Wuhan caused a worldwide panic. There are no specific antiviral therapies for COVID‐19. However, there are agents that were used during the severe acute respiratory syndrome (SARS) and Middle East respiratory syndrome (MERS) epidemics. We could learn from SARS and MERS. Lopinavir (LPV) is an effective agent that inhibits the protease activity of coronavirus. In this review, we discuss the literature on the efficacy of LPV in vitro and in vivo, especially in patients with SARS and MERS, so that we might clarify the potential for the use of LPV in patients with COVID‐19.

## Highlights

- LPV is an effective agent inhibiting coronavirus in vitro and animal studies.
- The treatment of LPV improved outcomes of SARS and MERS patients.
- LPV may be a potential treatment option for COVID‐19.

## 1 INTRODUCTION

In recent years, novel coronavirus infections have emerged periodically in various countries around the world. Severe acute respiratory syndrome coronavirus (SARS‐CoV) occurred in 2002, infecting 8422 people and causing 916 deaths during the epidemic.^1^ Middle East respiratory syndrome coronavirus (MERS‐CoV) was first identified in 2012.^2^ At the end of December 2019, a total of 2499 laboratory‐confirmed cases of Middle East respiratory syndrome (MERS), including 861 associated deaths were reported globally.^3^ At the end of 2019, novel coronavirus pneumonia (NCP) emerged in Wuhan and had spread rapidly. The pathogen was confirmed new coronavirus, which was officially named coronavirus disease‐19 (COVID‐19) by the World Health Organization (WHO). As of February 21, 2020, a total of 76 395 confirmed cases have been reported, and 2 348 patients are reported to have died. Currently, there is no specific antiviral treatment for COVID‐19. Therefore, identifying drug treatment options as soon as possible is critical for the response to the COVID‐19 outbreak.

SARS‐CoV, MERS‐CoV, and COVID‐19 belong to the same genera of CoV and all are beta‐CoV. COVID‐19 shares 79.5% sequence identity with SARS‐CoV.^4^ Therefore, the existing treatment LPV for SARS and MERS may be helpful for developing COVID‐19 therapeutics.

Proteinase is a key enzyme in CoV polyprotein processing. In recent years, research on SARS‐CoV and MERS‐CoV protease inhibitors has been carried out in vitro and in vivo. Lopinavir (LPV) is a proteinase inhibitor. Both peak (9.6 µg/mL) and trough (5.5 µg/mL) serum concentrations of LPV inhibit SARS‐CoV.^5^ LPV also blocks a post‐entry step in the MERS‐CoV replication cycle.^6^ Ritonavir (RTV) inhibits the CYP3A‐mediated metabolism of LPV, thereby increasing the serum concentration of LPV. Lopinavir/ritonavir (LPV/r) is a combination of lopinavir and ribavirin. The antiviral activity of LPV/r is similar to that of LPV alone, suggesting that the effect is largely driven by LPV.^7^, ^8^ In this review, we analyze the efficacy of LPV or LPV/r in patients with SARS‐CoV and MERS‐CoV, which can be a useful reference for COVID‐19 treatment option.

## 2 IN VITRO AND ANIMAL STUDIES

### 2.1 In vitro studies of SARS

An analysis of molecular dynamics simulations showed that the SARS‐CoV 3CLpro enzyme could be inhibited by the combination of lopinavir and ritonavir.^9^ A binding analysis of the main SARS coronavirus proteinase with LPV showed that half of lopinavir is left outside the catalytic site, and the efficacy of lopinavir may be poor.^10^ Another study showed that neither lopinavir nor ritonavir has an effect on the replication of SARS‐CoV.^11^

However, studies have revealed that lopinavir has antiviral activity. The 50% effective inhibitory concentration (EC_50_) of LPV for the plaque reduction assay is 6 µg/mL in the Vero cell line. The selectivity index (SI) of LPV is 8 to 32.^12^ In vitro activity against SARS‐CoV has been demonstrated for lopinavir at 4 µg/mL after 48 hours of incubation. Cytopathic inhibition has been achieved down to a concentration of lopinavir 1 µg/mL combined with ribavirin at 6.25 µg/mL and data suggested that this combination may be synergistic against SARS‐CoV in vivo.^13^

### 2.2 Animal studies of SARS

There have been some animal studies of SARS,^14^ however, no study of lopinavir or ritonavir has been performed.

### 2.3 In vitro studies of MERS

In an in vitro study, LPV inhibited MERS‐CoV‐induced cytopathic effect (CPE) with an EC_50_ of 8.0 μM (SI = 3.1), and a maximal protective effect (89% inhibition) was observed at a dose of 12 μM.^6^ However, an in vitro study showed that LPV was not effective. LPV showed a suboptimal EC_50_ in the initial cytopathic effect inhibition assay and was therefore not evaluated further.^15^ Another in vivo study of MERS showed that EC_50_ values generated for lopinavir and ritonavir were 11.6 and 24.9 μM with CC_50_ values > 50 μM, the SI for LPV and RTV was > 4.3 and > 2, respectively.^7^ Compared with remdesivir and interferon‐β (IFN‐β), LPV has inferior in vitro antiviral activity. RTV does not significantly enhance the antiviral activity of LPV in vitro.^7^

### 2.4 Animal studies of MERS

For the MERS‐CoV mouse model, prophylactic LPV/r combined with IFN‐β slightly reduced the viral loads.^7^ However, therapeutic LPV/r and IFN‐β improved pulmonary function, but failed to reduce viral replication and lung hemorrhaging. This in vivo evidence is suggestive of the potential for LPV/r to treat MERS‐CoV infections. When LPV/r was combined with IFN‐β, the antiviral activity (EC_50_ = 160 IU/mL) was indistinguishable from that of IFN‐β alone (EC_50_ = 175 IU/mL, *P* = .62). This suggests that the observed in vitro antiviral activity of the LPV/r‐IFN‐β combination against MERS‐CoV is dominated by IFN‐β when LPV/r is used at clinically relevant concentrations.

Chan et al^16^ explored the therapeutic potential of LPV/r and/or IFN‐β in common marmosets. Animals treated with LPV/r alone or in combination with interferonβ1b had better clinical scores, less weight reduction, and less pulmonary infiltrate than untreated animals. Furthermore, necropsied lung and extrapulmonary tissues from the treated group had lower mean viral loads than those from the control group.

The in vitro and animal studies of SARS and MERS are summarized in Table 1.

**Table 1:** A summary of in vitro and animal studies of SARS and MERS

SARS			MERS				
In vitro		Animal	In vitro				Animal
(9)	LPV/r could inhibit SARS‐CoV 3CLpro enzyme	No study of LPV/r included	(6)	LPV inhibit MERS‐CoV‐induced CPE with an EC_50_ of 8.0 μM (SI = 3.1)	(7)	Prophylactic LPV/r combine with IFN‐β	Reduces viral loads
(10)	The efficacy of LPV to SARS coronavirus could be poor		(15)	LPV were not effective in the initial cytopathic effect inhibition assay		Therapeutic LPV/r and IFN‐β	Improves pulmonary function
							Reduce virus replication
							Reduce lung hemorrhage
(11)	Neither LPV nor RTV had an effect on the replication of SARS‐CoV		(7)	The EC_50_ values generated for LPV were 11.6 μM (SI > 4.3). RTV does not significantly enhance the antiviral activity of LPV in vitro	(16)	LPV/r‐treated alone or in combination with interferon β1b	Better clinical scores
							Less weight reduction
							Less pulmonary infiltrate
(12)	The EC_50_ of LPV was 6 µg/mL (SI was 8‐32)						Lower viral loads in the lung
(13)	There is activity against SARS‐CoV with combination of LPV 1 µg/mL and RBV 6.25 µg/mL						

Abbreviations: CPE, cytopathic effect; EC50, 50% effective inhibitory concentration; INF, interferon; LPV, lopinavir; LPV/r, lopinavir/ritonavir; MERS‐CoV, Middle East respiratory syndrome coronavirus; RBV, ribavirin; RTV, ritonavir; SARS‐CoV, severe acute respiratory syndrome coronavirus; SI, selectivity index.

## 3 CLINICAL STUDIES

### 3.1 SARS

In a preliminary report, there were no deaths at 30 days after the onset of symptoms among 34 patients treated with LPV/r (400 mg ritonavir and 100 mg lopinavir) in combination with ribavirin initially, compared to 10% mortality in 690 patients taking only ribavirin. Twenty‐one percent of 33 patients who received LPV/r as a rescue therapy died, whereas 42% of 77 patients who received ribavirin alone died.^17^ However, these results were given only as a presentation, and no formal paper was published. Thus, this evidence is not credible.

A retrospective matched cohort study including 1052 SARS patients (75 treated patients and 977 control patients) showed that the addition of LPV/r as an initial treatment was associated with a reduced death rate (2.3%) and intubation rate (0%) compared with that in a matched cohort who received standard treatment (11.0% and 15.6%, respectively, *P* < .05).^18^ In addition, the rate and dose of pulsed methylprednisolone were decreased. These SARS patients were retrospectively matched with control subject. Matching was performed with respect to age, sex, the presence of comorbidities, lactate dehydrogenase level, and the use of pulsed steroid therapy. However, the mortality, oxygen desaturation, and intubation rates of the subgroup of patients who received lopinavir‐ritonavir as rescue therapy were not different from those in the matched cohort and patients who received an increased dose of pulsed methylprednisolone. This result suggests that the combination of lopinavir and ribavirin has a synergistic effect for the treatment of SARS; it may play an essential role in the early phase of the infection. The viral replication phase peaks around day 10.^19^ LPV/r use within this replication window decreases the peak viral load and the subsequent immune response.

Another retrospective matched cohort study of SARS patient also revealed that the rate of acute respiratory distress syndrome (ARDS) or death was significantly lower in the LPV/r combination treatment group (1/41, 2.4%) than the historical controls (32/111, 28.8%) on day 21.^13^ In addition, the LPV/r group had a progressive decrease in the viral load, an early rise in the lymphocyte count, a reduction in the cumulative dose of pulsed methylprednisolone, and fewer episodes of nosocomial infections. These findings show that LPV/r, when combined with ribavirin, may be an effective agent against SARS. The summary of the effects of LPV in SARS patients is shown in Table 2.

**Table 2:** A summary of the studies on the use of lopinavir therapy in SARS patients

Type	Patients	Ribavirin	Corticosteroids	Lopinavir/ritonavir		Outcome				References
Retrospective matched cohort study	1052	10‐14 d (2.4 g oral loading dose, followed by 1.2 g orally every 8 h, or 8 mg/kg intravenously every 8 h	21 d (starting dosage: hydrocortisone 100‐200 mg every 6‐8 h, or methylprednisolone 3 mg/kg/day, depending on the severity).	10 to 14 d of lopinavir 400 mg/ritonavir 100 mg orally every 12 h		Desaturation rate (SaO_2_ 95%) [%]	Intubation rate (%)	Death rate (%)	Mean pulsed methylprednisolone dose (g)	Chan et al^1^, ^18^
				Initial therapy (44)		68.2	0*	2.3*	1.6*	
				Control (634)		84.5	11.0*	15.6*	3.0*	
				Rescue therapy (31)		93.5	9.7	12.9	3.8*	
				Control (343)		92.1	18.1	14.0	3.0*	
Retrospective matched cohort study	152	14 d (4 g oral loading dose followed by 1.2 g orally every 8 h, or 8 mg/kg intravenously every 8 h	21 d (starting dosage: hydrocortisone 100‐200 mg every 6‐8 h, or methylprednisolone 3 mg/kg/day, depending on the severity).	14 d of lopinavir 400 mg/ritonavir 100 mg orally every 12 h		ARDS/death rate (%)	Viral load and lymphocyte count	Nosocomial infections (%)	Mean pulsed methylprednisolone dose (g)	Chu et al^13^
				Treatment group	Initial treatment	2.4*	Viral load↓ and lymphocyte count↑	0*	1.5 g*	
					Rescue treatment			27.6*	2.5 g*	
				Historical control group		28.8*	/	25.2*	2.0 g	

* Indicates a significant difference, *P* < .05.

### 3.2 MERS

A MERS patient who received LPV/r, ribavirin, and interferon had a resolution of viremia after 2 days of treatment.^20^ However, the patient eventually died from septic shock 2 months and 19 days after the initial diagnosis. Another 64‐year‐old MERS patient from Korea was also treated with LPV/r, ribavirin, and interferon. After 6 days of antiviral therapy, negative PCR result in the serum sample, sputum samples, and swab samples were achieved.^21^ The patient was discharged on day 13 of admission after achieving complete recovery. These two simple cases may show that LPV is effective against MERS. However, they do not exclude the possibility of other combination therapies being effective or spontaneous improvement occurring. The treatment effect of LPV/r against MERS is still controversial.

A retrospective study enrolled healthcare workers (HCWs) with high‐risk exposure to MERS‐CoV pre‐isolation pneumonia and revealed that an effective post‐exposure prophylaxis (PEP) strategy including LPV/r may limit the spread of infection.^22^ PEP therapy was associated with a 40% decrease in the risk of infection with no severe adverse events during treatment. PEP therapy was a significant factor that reduced the risk of MERS‐CoV infection in HCWs. This finding may indirectly reflect the antiviral effect of LPV/r. Moreover, a combination regimen of interferon + ribavirin + LPV/r was recommended officially for MERS therapy in Korea, where MERS began to spread in 2015.^23^ Without randomized controlled trials, determining treatment is difficult due to patient and treatment variability as well as a lack of appropriately matched controls. The combination of LPV/r and interferon was considered in a randomized control trial in Saudi Arabia.^24^ Enrollment began in November 2016 and the results are not yet available. The summary of LPV research in MERS patients is shown in Table 3.

**Table 3:** A summary of the studies on the use of lopinavir therapy in MERS patients

Type	Patients	Ribavirin	Interferon	Intubation	Lopinavir/ritonavir	Outcome	References
Case report	1 patient, 64 M	7 d, 2 g oral loading dose followed by 1.2 g every 8 h per day orally	Pegylated interferon 180 µg/0.5 mL, subcutaneously on day 4 of admission	No	7 d, LPV/r (400/100 mg twice daily), per oral	Fever was absent. PCR results of serum samples, sputum samples, and swab samples were all negative 6 d after antiviral therapy. The patient was discharged on day 13 of admission after achieving complete recovery.	Kim et al^21^
Case report	1 patient, 69 M	ribavirin (2000 mg p.o. loading dose, followed by 1200 mg p.o. every 8 h for 8 d)	Pegylated interferon 180 g subcutaneously once per week for 12 d	yes	10 d, LPV/r (400/100 mg twice daily), per oral	Viremia resolved after 2 d of treatment but ultimately died from septic shock.	Spanakis et al^20^
Retrospective matched cohort study	43 healthcare workers (HCWs)	ribavirin (loading dose of 2000 mg followed by 1200 mg every 8 h for 4 d and then 600 mg every 8 h for 6‐8 d)	/	/	11‐13 d, LPV/r (400/100 mg twice daily), per oral	Therapy was initiated between days 1 and 3 after the last unprotected exposure to a MERS patient.	Park et al^22^
						PEP therapy was associated with a 40% decrease in the risk of infection. There were no severe adverse events during PEP therapy.	
Randomized controlled trial	/	/	IFN‐β1b 0.25 mg/mL SQ on alternative days for 14 d	/	14 d, LPV/r (400/100 mg twice daily), per oral	Results are not yet published.	Arabi et al^24^

Abbreviations: IFN, interferon; LPV/r, lopinavir/ritonavir; MERS, Middle East respiratory syndrome; PCR, polymerase chain reaction; PEP, post‐exposure prophylaxis.

### 3.3 COVID‐19

There are no reported in vitro studies of COVID‐19. Four patients with COVID‐19 were given antiviral treatment including LPV/r. After treatment, three patients showed significant improvement in pneumonia‐associated symptoms, two of whom were confirmed to be COVID‐19 negative and discharged, and one of whom was negative for the virus at the first test.^25^ This study shows the positive effects of LPV/r therapy. Two reviews, including a Chinese review and communication showed that LPV may be drug treatment option for COVID‐19.^26^, ^27^ However, a retrospective study enrolled 134 NCP patients revealed that there is no significant difference between LPV/r‐treated group (n = 52), Abidol‐treated group (n = 34), and control group (n = 48) in improving symptom or in reducing viral loads.^28^ The negative rate of COVID‐19 nucleic acid on the 7 day was 71.8%, 82.6%, and 77.1%, respectively (*P* = .79). The efficacy of LPV/r antiviral treatment warrants further verification in future studies. Nine randomized controlled trials of LPV/r in patients with COVID‐19 have been registered in China up to February 22 (Table 4). Currently, the combination of LPV/r is a recommended antiviral regimen in the latest version of the Diagnosis and Treatment of Pneumonia Caused by COVID‐19 issued by the National Health Commission of the People's Republic of China.

**Table 4:** Clinical trials of lopinavir (LPV) in patients with 2019‐new coronavirus (2019‐nCoV) registered in China (up to February 22)

Registration number	Registration date	Institution	Title	Enrolment date
ChiCTR2000029308	2020/1/23	Wuhan Infectious Diseases Hospital	A randomized, open‐label, blank‐controlled trial for the efficacy and safety of lopinavir‐ritonavir and interferon‐alpha 2b in hospitalized patients with novel coronavirus pneumonia (COVID‐19)	2020/1/10
ChiCTR2000029468	2020/2/2	Sichuan People's Hospital, Sichuan Academy of Medical Sciences	A real‐world study for lopinavir/ritonavir (LPV/r) and emtritabine (FTC)/tenofovir alafenamide fumarate tablets (TAF) regimen in the treatment of 2019‐nCoV pneumonia (novel coronavirus pneumonia, NCP)	2020/2/1
ChiCTR2000029539	2020/2/3	Tongji Hospital, Huazhong University of Science and Technology	A randomized, open‐label study to evaluate the efficacy and safety of lopinavir‐ritonavir in patients with mild 2019‐nCoV pneumonia (novel coronavirus pneumonia, NCP)	2020/2/4
ChiCTR2000029541	2020/2/3	Zhongnan Hospital of Wuhan University	A randomized, open, controlled trial for darunavir/cobicistat or lopinavir/ritonavir combined with thymosin a1 in the treatment of 2019‐nCoV pneumonia (novel coronavirus pneumonia, NCP)	2020/2/10
ChiCTR2000029548	2020/2/4	The First Affiliated Hospital, Zhejiang University School of Medicine	Randomized, open‐label, controlled trial for evaluating of the efficacy and safety of Baloxavir Marboxil, Favipiravir, and Lopinavir‐Ritonavir in the treatment of 2019‐nCoV pneumonia (novel coronavirus pneumonia, NCP) patients	2020/2/4
ChiCTR2000029573	2020/2/4	The First Affiliated Hospital of Medical College of Zhejiang University	A multicentered, randomized, open‐label, positive‐controlled trial for the efficacy and safety of recombinant cytokine gene‐derived protein injection combined with abidole, lopinavir/litonavir in the treatment of 2019‐nCoV pneumonia (novel coronavirus pneumonia, NCP) patients	2020/2/6
ChiCTR2000029603	2020/2/6	The First Affiliated Hospital of Zhejiang University School of Medicine	A randomized, open‐label, multicenter clinical trial evaluating and comparing the safety and efficiency of ASC09/ritonavir and lopinavir/ritonavir for confirmed cases of 2019‐nCoV pneumonia (novel coronavirus pneumonia, NCP)	2020/2/6
ChiCTR2000029741	2020/2/11	The Fifth Affiliated Hospital Sun Yat‐Sen University	Efficacy of chloroquine and lopinavir/ritonavir in mild/general novel coronavirus (CoVID‐19) infections: a prospective, open‐label, multicenter randomized controlled clinical study	2020/2/12
ChiCTR2000029759	2020/2/12	The Second Affiliated Hospital of Chongqing Medical University	A multicenter, randomized, open‐label, controlled trial for the efficacy and safety of ASC09/ritonavir compound tablets and lopinavir/ritonavir (Kaletra) and Arbidol tablets in the treatment of novel coronavirus pneumonia (COVID‐19)	2020/2/15

## 4 DISCUSSION

Currently, there are no FDA‐approved treatments for any human CoV infection. Upon the emergence of SARS‐CoV and MERS‐CoV, patients were administered off‐label antivirals. Most in vitro studies have shown that SARS‐CoV could be inhibited by LPV and that the EC_50_ of LPV is acceptable. Furthermore, two retrospective matched cohort studies of SARS patients revealed that LPV/r plays an essential role in the clinical outcome, especially in the early stage. LPV/r‐treatment alone or in combination with interferon had improved clinical outcomes in experiments involving common marmosets and in some MERS patient. However, we need to wait for more clinically valid evidence to confirm the positive value of LPV for COVID‐19 treatment.

Although most of the data indicate the efficacy of LPV, adverse reactions should be kept in mind. Diarrhea, nausea, and asthenia are the most frequently reported reactions in patients receiving LPV therapy.^5^ Elevated total bilirubin, triglyceride, and hepatic enzyme levels have also been reported.^20^, ^21^ A retrospective study of MERS showed that the most common symptoms and laboratory tests of LPV/r PEP were diarrhea (40.9%), nausea (40.9%), stomatitis (18.2%), fever (13.6%), anemia (45.0%), leukopenia (40.0%), and hyperbilirubinemia (100%).^22^ However, the symptoms and laboratory tests returned to normal after LPV therapy ceased.

The protease inhibitor LPV could be an effective treatment based on the experience accumulated from the SARS and MERS outbreaks. The treatment of CoV patients with LPV/r improved their outcomes. LPV/r may be a potential treatment option for COVID‐19. Additional studies are needed to gain further insights into the origin, tropism, and pathogenesis of COVID‐19.

## CONFLICT OF INTERESTS

The authors declare that there are no conflict of interests.

## AUTHOR CONTRIBUTIONS

TTY and JDQ wrote the article, include the concept of this article, definition of intellectual content, and data acquisition; WYZ contributed for data acquisition; YW and GQW designed and reviewed the manuscript for its intellectual content.
